# EGF-Induced Bronchial Epithelial Cells Drive Neutrophil Chemotactic and Anti-Apoptotic Activity in Asthma

**DOI:** 10.1371/journal.pone.0072502

**Published:** 2013-09-11

**Authors:** Mohib Uddin, Laurie C. Lau, Grégory Seumois, Pandurangan Vijayanand, Karl J. Staples, Dinesh Bagmane, Victoria Cornelius, Paul Dorinsky, Donna E. Davies, Ratko Djukanović

**Affiliations:** 1 Academic Unit of Clinical and Experimental Sciences and the NIHR Southampton Respiratory Biomedical Research Unit, Southampton University Faculty of Medicine, Sir Henry Wellcome Laboratories, Southampton University Hospital, Southampton, United Kingdom; 2 GlaxoSmithKline, Respiratory Medical Development Center, Research Triangle Park, Durham, North Carolina, United States of America; Northwestern University Feinberg School of Medicine, United States of America

## Abstract

Chronic damage and repair of the bronchial epithelium are features of asthma. We have previously reported that *ex vivo* stimulation of normal bronchial epithelial cells with epidermal growth factor (EGF), a key factor of epithelial repair, enhances the mechanisms of neutrophil accumulation, thereby promoting neutrophil defences during acute injury but potentially enhancing inflammation in chronic airway diseases. We have now sought to (i) determine whether this EGF-dependent pro-neutrophil activity is increased in asthma, where EGF and its epithelial receptor are over-expressed, and (ii) elucidate some of the mechanisms underlying this asthmatic epithelial-neutrophil interaction. Primary bronchial epithelial cells (PBEC) from healthy subjects, mild asthmatics and moderate-to-severe asthmatics (Mod/Sev) were stimulated with EGF, a model that mimics a repairing epithelium. Conditioned culture media (EGF-CM) were assessed for neutrophil chemotactic and anti-apoptotic activities and inflammatory mediator production. EGF induced the epithelium to produce soluble mediators with neutrophil chemotactic (p<0.001) and pro-survival (p = 0.021) activities which were related to the clinical severity of asthma (trend p = 0.010 and p = 0.009, respectively). This was associated with enhanced IL-6, IL-8, GM-CSF and TNF-α release, and cytokine-neutralising experiments using EGF-CM from Mod/Sev asthmatics demonstrated a role for GM-CSF in neutrophil survival (p<0.001). Pre-treatment of neutrophils with specific inhibitors of the myeloid-restricted class I phosphatidylinositol-3-OH kinase (PI(3)K) isoforms showed that the EGF-CM from Mod/Sev asthmatics depended on the γ (p<0.021) but not δ isoforms, while neutrophil survival required multiple class I PI(3)Ks. The EGF-induced chemotactic, but not pro-survival activity, involved RhoA signaling in neutrophils (p = 0.012). EGF whose activity is upregulated in asthma induces *ex vivo* the epithelium from asthmatic patients to produce pro-neutrophil activities; these are related to asthma severity and, in moderate-to-severe asthmatics, involves class IB PI(3)Kγ signaling, providing a potential therapeutic target for neutrophilic forms of asthma.

## Introduction

Neutrophilic airway inflammation is a common feature of severe chronic asthma [1], [2], [3], [4] shown to be insensitive to glucocorticoids (GCs) [5], [6], but the mechanisms which regulate the accumulation of neutrophils within the inflamed airways are still poorly understood. Several studies in asthma have reported raised concentrations of factors in the airways that have the potential to chemoattract neutrophils and inhibit their apoptosis including, interleukin (IL)-8 [3], [7], IL-6 [8],[9],[10], granulocyte-macrophage colony-stimulating-factor (GM-CSF) [8], [9] and tumour necrosis factor (TNF)-α [11], [12]. A clear link between raised levels of these factors and enhanced neutrophil chemotactic and anti-apoptotic activity in asthma has yet to be established.

Delayed apoptosis, which is responsible for increased neutrophil longevity in tissues is thought to impede the resolution of airway inflammation [13]. We have recently detected significant neutrophil anti-apoptotic activity in the epithelial lining fluid of severe asthmatic patients with sputum neutrophilia in whom far fewer apoptotic neutrophils were observed [4], but have been unable to identify the responsible factor (s). In an earlier study using the bronchial 16HBE cell line and primary bronchial epithelial cells (PBECs) from healthy individuals, we showed that *ex vivo* the bronchial epithelium produces an array of neutrophil chemotactic factors, IL-8, GM-CSF, TNF-α and LTB_4_ [14]. In the same study, we also showed that epidermal growth factor (EGF), an important factor of epithelial repair, enhanced the *ex vivo* production of these chemotactic factors by the epithelium. In addition to regulating airway mucosal injury and repair responses, EGF has also been shown to contribute to airway wall remodeling [15], lung inflammation [15], [16], [17] and airway dysfunction in a chronic mouse model of allergic lung inflammation [18]. Over-expression of EGF receptor (EGFR) and its ligands (*i.e.* EGF, amphiregulin, heparin-binding EGF-like growth factor) has been observed in the airways of adult [19], [20], [21], [22] as well as paediatric asthmatics [23], [24], with levels of EGF and amphiregulin being significantly elevated following acute exacerbations in the latter patient population [25], [26]. This suggests that the pathobiology of asthma involves and may in fact result, in part, from EGFR-mediated dysregulation of injury-repair responses in the airway mucosa. Consistent with this concept, immunostaining for the tyrosine-kinase linked EGFR is increased in the asthmatic bronchial epithelium in relation to disease severity and correlates with IL-8 expression and neutrophil numbers [17]. *Ex vivo* stimulation of airway epithelial cells with EGF induces production of IL-8 [14], [17], [27] and this response is insensitive to GCs in airway epithelial cells from asthmatics [17], [28]. Together with the recent identification of functional variants in genes linking epithelial damage to the adaptive immune system [29], these studies point to an important role for the airway epithelium in the pathogenesis of asthma [15]. However, a direct link between the observed effects of EGF on endogenous mediator production by the epithelium and neutrophil accumulation has not been established, nor is there evidence that this is a feature of clinical asthma.

In the current study, we tested the hypothesis that EGF plays an important role in activating the bronchial epithelium of asthmatic patients by up-regulating the release of mediators that recruit neutrophils and/or inhibit their apoptosis in the airways. We also hypothesised that this pro-neutrophilic activity increases in relation to the clinical severity of asthma and thus provides a potential mechanism for the unresolved airway neutrophilia observed in more severe forms of this disease. To test this concept, we applied our translational *ex vivo* model which uses primary epithelial cell (PBEC) culture established from bronchoscopic brushings of control subjects and asthmatic patients [19], [30]. Cells were either left in culture unstimulated or exposed to EGF, thus mimicking an *in vivo* situation where the asthmatic epithelium sustains injury and this is followed by repair, features that are central to the pathogenesis of asthma. Using the conditioned media (CM) from these cultures to assess their effects on neutrophil chemotaxis and survival, we sought to determine whether, as part of an acute insult, such as virus infection, associated with ∼80% of asthma exacerbations in paediatrics [31], or a chronic injury-repair process, EGF can activate the asthmatic epithelium in a way that modulates neutrophilic inflammatory responses and whether this is related to asthma severity. Finally, in order to improve understanding of the signaling pathways in neutrophils responding to stimuli from an EGF-activated epithelium, we studied the effect of inhibiting phosphatidylinositol-3-OH kinase (PI(3)K) signaling on any induced neutrophil activity, focusing on the myeloid-restricted class I PI(3)K isoforms, γ and δ [32]. Despite convincing preclinical evidence that these PI(3)Ks are involved in driving neutrophilic and allergic-type pulmonary inflammation *in vivo* [33], [34], [35], [36], little is known about how these kinases and their downstream signaling in neutrophils are regulated by epithelium-derived mediators in the context of clinical asthma.

## Methods

### Subjects

Twenty-eight non-smoking adult volunteers (8 steroid-naïve mild asthmatics (MA), 11 moderate-to-severe asthmatics (Mod/Sev) and 9 non-atopic healthy control subjects (HC)) took part in the study (see Table 1). Subjects with mild asthma had symptoms >2 times/week, with forced expiratory volume in 1 sec (FEV_1_) >80% of predicted and used short-acting inhaled β_2_-agonists as needed for symptom relief. Subjects with moderate asthma were treated with low-dose inhaled GCs and prn inhaled short-acting β_2_-agonists. Subjects with severe asthma were taking regular oral or high-dose inhaled GCs and a long-acting inhaled β_2_-agonist, but despite that had FEV_1_ <80% of predicted. The moderate and severe asthmatics were analysed as one group (Mod/Sev). The definition of asthma severity was in keeping with Global Initiative for Asthma (GINA) 2010 criteria http://www.ginasthma.org (s.1). Atopy was assessed by skin prick tests for common allergens. The age-matched healthy controls were non-atopic, had no history of smoking or respiratory symptoms suggestive of asthma and all had FEV_1_ >90% of predicted.

**Table 1:** Demographic and clinical characteristics of non-smoking patients with asthma and non-atopic healthy controls.

	Healthy Controls (*n = 9*)	Mild Asthmatics (*n = 8*)	Moderate/Severe Asthmatics (*n = 11*)
Age (*yr*)	43.67±6.10	36.50±4.52	42.55±5.02
Gender (*M/F*)	5/4	4/4	8/3
Atopy, *n*	*None*	8	11
FEV_1_ (*% predicted*)	106.96±3.29	87.02±4.73**^††^**	92.53±6.52
Postbronchodilator FEV_1_ (*L*)	3.49±0.32	3.05±0.11	3.31±0.29
ICS dose (*μg/d*)	*None*	*None*	431.82±90.52
β_2_ agonist dose (*μg/d*)	*None*	175.0±16.37	163.64±40.50

Data are expressed as mean ± SEM and statistical significance by unpaired *t*-test, **††**p<0.01 *versus* MA and HC; one-way ANOVA with post-hoc test and Bonferroni correction (p = 0.05).

The study was approved by the South and West Local Research Ethics Committee, Southampton, U.K. and all subjects gave their full informed written consent in accordance with the Declaration of Helsinki.

### Sampling and culture of primary bronchial epithelial cells

Primary bronchial epithelial cells were obtained by gentle brushing of the third to fourth generation bronchi during fibreoptic bronchoscopy as previously described [14], [30]. The epithelial cell were cultured in hormonally supplemented bronchial epithelial growth medium (BEGM) (Clonetics, San Diego, CA, USA) in collagen-coated tissue culture flasks (Gibco-BRL, Paisley, UK) at 37°C in 5% (v/v) CO_2_. At passage 2, approximately 4×10^5^ cells/well were seeded into 24-well culture plates and grown to 70% confluency. Cells were first rendered quiescent for 24 h in serum-free medium (SFM) consisting of BEBM basal medium (Clonetics, USA), supplemented with 1% (v/v) insulin/transferrin/sodium selenite (Sigma, Poole, UK) and 0.3% (v/v) bovine serum albumin (BSA) and were then cultured further for 24 h in SFM containing EGF (10 ng/ml) or SFM alone. This generated EGF-conditioned media (EGF-CM) and basal-conditioned media (basal-CM), respectively.

### Analysis of bronchial-epithelial-derived mediators

Basal-CM and EGF-CM from all 3 subject groups were analysed for IL-6, IL-8, TNF-α and GM-CSF using a combination of Luminex-based multiplex assays (Bio-Rad) and ELISA (R&D systems) according to manufacturer's instructions.

### Assessment of bronchial epithelial cell proliferation

Epithelial cell number was determined by uptake of methylene blue [37]. Subconfluent epithelial cells were seeded into 24-well plates at 2.5×10^4^ cells/well in complete BEGM medium and allowed to adhere at 37°C. After 6 h, the medium was changed to SFM, serum starved overnight, and then stimulated with SFM or EGF (10 ng/ml) for 24 h. Monolayers were fixed with 4% formaldehyde in 0.9% NaCl solution (20°C for 1 h) and stained with 1% methylene blue in 10 mM disodium tetraborate (pH 8.5) for 30 min and then washed again. Bound methylene blue was eluted with 1% HCl in ethanol and cell number determined by measuring the absorbance at 650 nm (A_650_) with a microplate spectrophotometer (MultiScan Ascent, Affinity Sensors, Cambridge, UK). A_650_ was directly proportional to the epithelial cell number over the range of cell densities. The stimulation index was calculated by dividing the mean number of epithelial cells exposed to EGF (10 ng/ml, 24 h) by the mean number of cells incubated with SFM alone.

### Isolation of neutrophils from healthy human volunteers

Neutrophils were purified from peripheral blood by dextran sedimentation and centrifugation through plasma/Percoll gradients as previously described [14]. All neutrophil donors were healthy and non-atopic so as to avoid eosinophils whose numbers may be raised in atopic/asthmatic patients. Isolated neutrophils were routinely >96% pure and >98% viable as assessed by cytospin and trypan blue exclusion, respectively. Endotoxin-free reagents were used throughout to prevent inadvertent neutrophil priming.

### Assessment of neutrophil chemotaxis

The chemotactic activity of basal-CM or EGF-CM on neutrophil migration was measured in a fluorescence-based Neuroprobe ChemoTX© microplate as described [14]. Briefly, the lower wells of the microplate were filled with either 31 µl of SFM, basal-CM or EGF-CM. Calcein-labeled neutrophils (3×10^5^ cells/ml) were loaded to the upper aspect of the membrane and allowed to migrate for 60 min (37°C in 5% CO_2_). After washing, transmigrated neutrophils were quantified by measuring fluorescence (485 nm excitation, 530 nm emission wavelengths) using a fluorescence plate reader.

### Analysis of neutrophil apoptosis

Purified neutrophils (5×10^6^ cells/ml) were incubated in CM for 20 h after which apoptosis was assessed by flow cytometry (FACSCalibur, BD Biosciences) using fluorescein isothiocyanate-labelled Annexin-V binding and propidium iodide (Annexin-V^FITC^/PI) staining. Ten thousand gated events were acquired for each sample and neutrophil apoptosis was expressed as a percentage of apoptotic cells in relation to the total number of counted neutrophils. This method was previously validated by comparison with the gold standard microscopic method which assesses neutrophil apoptosis using nucleus morphological criteria [4], [38].

Further details of the experimental methods are provided in File S1.

### Statistics

Data were assessed for normality and where appropriate presented as mean ± SEM. The difference across three or more groups (of independent experiments) was investigated using one-way analysis of variance (ANOVA). Comparisons made within a participant sample were analysed using a paired Student *t*-test for two groups or a repeated one-way ANOVA when there were three or more groups. When the results from the ANOVA analysis suggested evidence of a difference in mean values (p<0.05) post hoc tests, using Bonferroni adjustment to correct for multiple comparisons, were performed to determine where these differences lay. Trend across the subject groups (severity of asthma) was assessed by linear trend (regression) analysis. For data that were not normally distributed, pair-wise comparisons were made using the Mann-Whitney test whereas the Kruskal-Wallis test was performed to determine if there were significant differences between three or more groups. Trend across the subject groups was assessed using the Cuzick's non-parametric test. Analyses were performed using Stata version 11 (StataCorp) graphs were constructed using Prism (GraphPad). A p value <0.05 was considered significant.

## Results

### Pilot experiments to establish the bronchial epithelial culture *ex vivo* model

#### Assessment of epithelial cell proliferation

**Figure 1:**
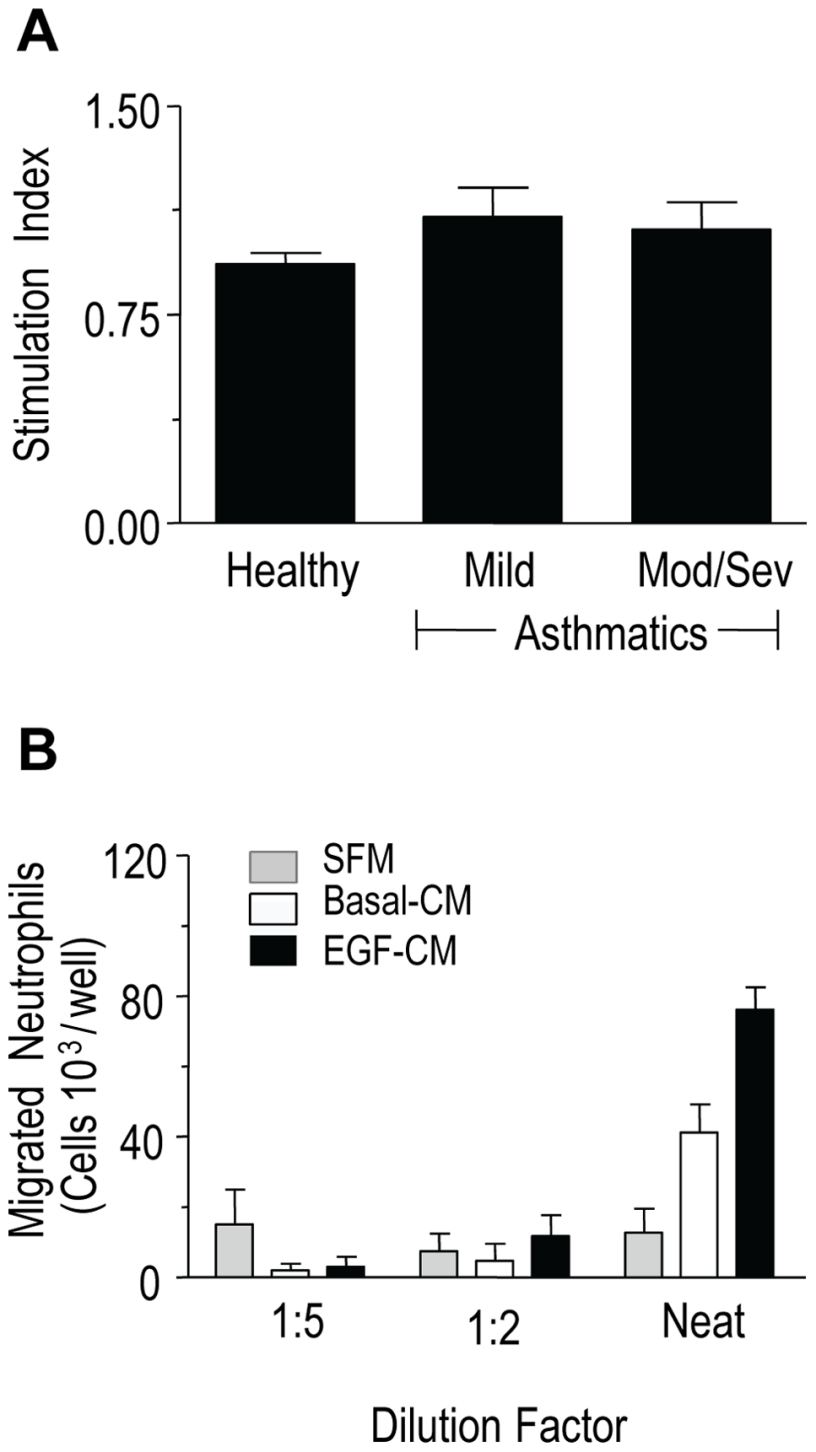
Effects of EGF on primary bronchial epithelial cell (PBEC) proliferation and mediating neutrophil chemotaxis. (A) PBECs were exposed to EGF and proliferation was assessed using a colorimetric assay based on methylene blue uptake and release (*see Results section; pilot experiments*). The stimulation index derived by dividing the methylene blue uptake of cells exposed to EGF (10 ng/ml, 24 h) by the uptake of unexposed cells were <1 in all subject groups, suggesting that, EGF did not increase the numbers of epithelial cells (*n* = 3–5 donors from each group). (B) Dose-dependency of chemotactic responses to PBEC conditioned media. PBECs were stimulated with EGF (10 ng/ml) to generate EGF-CM or left untreated (Basal-CM). Neutrophil chemotaxis induced by the CM was compared to SFM as described in *Methods*. The chemotactic activity was concentration-dependent with a maximal effect observed when using neat CM (*n* = 3 donors).

In order to assess whether any observed effects of EGF-activated PBECs on neutrophil chemotaxis or anti-apoptotic survival are due to an increase in the numbers or activity of existing epithelial cells following EGF stimulation, an assessment of proliferation of PBECs exposed to EGF was first performed using a colorimetric assay based on methylene blue uptake and release (see File S1). This showed that epithelial cell numbers were unchanged by EGF stimulation for 24 h when compared with SFM alone, implying that any observed changes in pro-neutrophil activity induced by EGF would be due to increased secretory activity of non-proliferating epithelial cells (Figure 1A).

#### Selection of dilution of PBEC conditioned medium for use in neutrophil experiments

Chemotactic dose-responses are usually bell-shaped and before proceeding with the full analysis of PBEC conditioned media, pilot experiments were conducted to assess whether the chemotactic responses were optimal to neat EGF-CM or 1∶2 and 1∶5 dilutions thereof. This showed that maximum chemotactic responses were observed with neat CM (Figure 1B). Thus, neat CM samples were used in all subsequent experiments.

### Assessment of pro-neutrophil activity in PBEC cultures from asthmatic and control subjects

**Figure 2:**
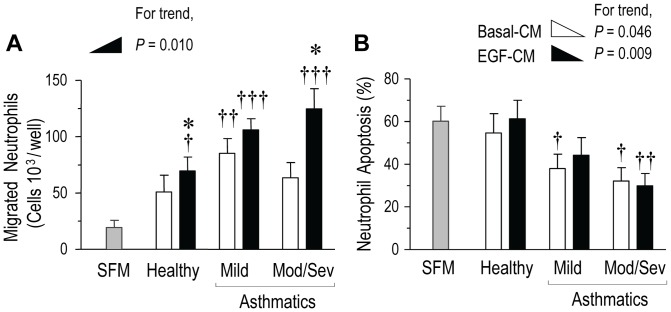
Neutrophil chemotactic and anti-apoptotic activity generated by EGF-induced bronchial epithelial cells. Conditioned media (CM) from 9 healthy control subjects, 8 mild and 11 Mod/Sev asthmatics were assessed for neutrophil chemotactic and anti-apoptotic activity as described in *Methods.* (A) Neutrophil chemotaxis was significantly induced by stimulation of PBECs with EGF in HC (p = 0.021) and Mod/Sev asthma (p = 0.010) but not in MA (0.093). The chemotactic activity in EGF-CM was related to the clinical severity of asthma (linear contrast test p = 0.010) (*n* = 8 donors). (B) Neutrophil apoptosis was assessed after 20 h of culture by FACS analysis of Annexin-V^FITC^/PI staining and both basal and EGF-CM from the asthma-derived PBECs, but not from HC, contained significant neutrophil anti-apoptotic activity when compared to SFM (MA, p = 0.047 and Mod/Sev, p = 0.014; HC, p = 0.603, respectively). The anti-apoptotic activity in basal-CM and EGF-CM increased in relation to disease severity (p = 0.046 and 0.009, respectively, as assessed by trend analysis), (*n* = 7 donors). Repeated one-way ANOVA with post-hoc Bonferroni; ^†^p<0.05, ^††^p<0.01 and ^†††^p<0.001 compared to SFM-treated neutrophils. *p<0.05 *vs.* basal-CM.

Having validated the model for PBECs, we proceeded to assess the chemotactic and neutrophil survival-enhancing activities in the CM from PBECs derived from the three subject groups (HC, MA and Mod/Sev). Unstimulated PBECs produced increased neutrophil chemotactic activity (as measured in basal-CM) in MA subjects which was greater than the chemotactic responses to SFM alone (ANOVA p = 0.007; Bonferroni post-hoc test p = 0.299 in HC, 0.004 in MA and 0.080 in Mod/Sev asthma, Figure 2A). By comparison with SFM, EGF-CM contained significantly higher chemotactic activity in all subject groups (ANOVA p<0.001, with Bonferroni post-hoc test p values of 0.036 for HC and <0.001 for both MA and Mod/Sev asthmatics). In HC and Mod/Sev, but not the MA subjects, stimulation of PBECs with EGF enhanced the chemotactic activity when compared to basal-CM (p = 0.021 in HC, 0.093 in MA and 0.010 in Mod/Sev asthma, Table S1); the EGF-CM activity was related to clinical asthma severity as shown by an increasing trend (linear contrast test p = 0.010) (Figure 2A).

A total of 60.31 ± 6.88% (range 42.35–80.51%) of the neutrophils underwent constitutive (spontaneous) apoptosis in culture medium by 20 h. Basal and EGF-CM from all the PBEC cultures from asthmatic, but not from healthy control subjects, contained augmented neutrophil survival activity as shown by the reduced spontaneous apoptosis of neutrophils in culture in CM when compared to SFM (ANOVA p = 0.041; Bonferroni post-hoc p values of 0.603 for HC, 0.047 for MA and p = 0.014 for Mod/Sev) (Figure 2B). The anti-apoptotic activity also showed an increasing trend with asthma severity (linear contrast tests, basal-CM p = 0.046 and EGF-CM p = 0.009) (Figure 2B), as observed previously with airway epithelial lining fluid (sampled by sputum induction) from severe asthmatics [4], but there was no additional EGF-induced effect when compared to anti-apoptotic responses mediated by unstimulated PBECs (Table S1).

### EGF induces production of cytokines/chemokines in PBEC cultures

**Figure 3:**
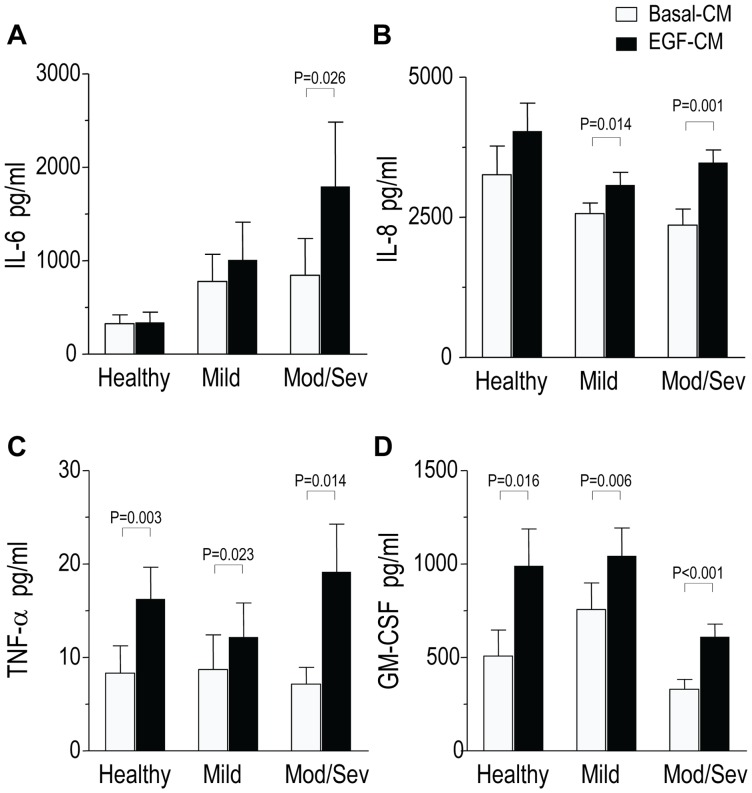
Induction of soluble mediator release by the asthmatic and normal bronchial epithelial cells in response to EGF stimulation. In Mod/Sev asthmatics (*n* = 11 patients), a significant induction of all four pro-inflammatory cytokines was seen, whilst in MA subjects (*n* = 8 patients), three of four mediators were induced; in the HC subjects (*n* = 9 donors), only two mediators were induced significantly. Linear contrast testing did not show any disease related trend in mediator production.

The CM were analysed for production of pro-neutrophil factors IL-6, IL-8, GM-CSF and TNF-α by PBECs. Analysis of basal-CM and EGF-CM showed no differences between the subject groups and there was no linear trend. While IL-8, GM-CSF and TNF-α were induced by EGF in PBECs in all subject groups (Figure 3A-D), IL-6 was induced significantly only in Mod/Sev asthmatics (Figure 3A). While significant induction of all four mediators was seen in Mod/Sev subjects and three of four mediators were induced in MA subjects, in the HC subjects only two mediators were induced significantly (Figure 3C and D). In contrast, the concentrations of the lymphotactic chemokines, TARC/CCL17 and MDC/CCL22 were undetectable or low in most samples in all three subject groups (data not shown).

### Characterization of chemotactic and anti-apoptotic activity in PBEC-CM derived from moderate to severe asthma patients

**Figure 4:**
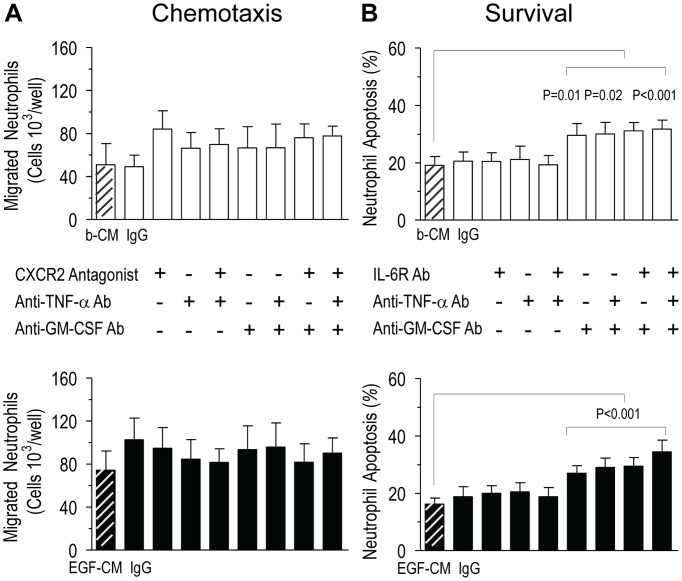
Characterization of neutrophil chemotactic and anti-apoptotic activity in PBEC-CM from Mod/Sev asthmatics. A) Neutralizing monoclonal antibodies (mAb) against TNF-α or GM-CSF (100 μg/ml or their isotype-matched IgG controls) and the CXCR2 antagonist, SB-225002 (100 nM) did not affect basal-CM (*open bars*) or EGF-CM- (*solid bars*) mediated neutrophil migration (*n* = 8 donors). (B) In neutrophil apoptosis studies, neutralization of GM-CSF activity alone or in combination with TNF-α or IL-6R blockade inhibited basal-CM and EGF-CM-induced survival (*n* = 7 donors). Data represent mean ± SEM analysed using repeated one-way ANOVA with post-hoc Bonferroni correction.

Having observed clearly augmented pro-inflammatory mediator responses to EGF-stimulation of PBECs from patients with Mod/Sev asthma, their relative roles were analysed using neutralising monoclonal antibodies (mAb) against GM-CSF and TNF-α and IL-6R (for neutrophil survival) and the selective antagonist for the CXCR2 receptor, SB-225002 (for neutrophil chemotaxis) at concentrations validated previously [14]. This showed that the neutrophil chemotactic activity in basal-CM or EGF-CM could not be inhibited by any of these blocking agents (Figure 4A). In contrast, anti-GM-CSF mAb, applied alone or together with either anti-TNF-α or anti-IL-6R mAbs, blocked the pro-survival effects of both basal-CM (repeated one-way ANOVA compared to untreated basal-CM p<0.001) and EGF-CM (repeated one-way ANOVA compared with untreated EGF-CM p<0.001; for p values for individual blocking agents see Figure 4B). This suggested that GM-CSF is an important regulator of neutrophil survival in Mod/Sev asthmatic PBECs.

### Activation of class I PI(3)Ks and downstream signaling in neutrophils

**Figure 5:**
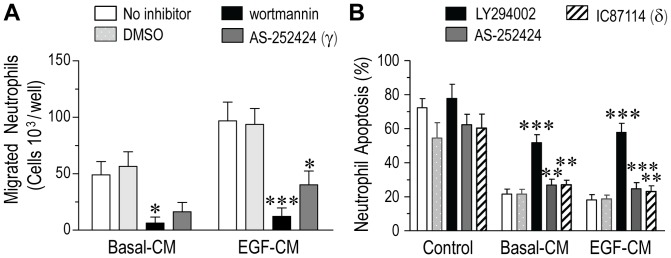
Activation of class I PI(3)Ks during neutrophil chemotaxis and survival induced by PBEC-CM from Mod/Sev asthmatics. (A) Pretreatment of neutrophils with wortmannin (100 nM, 30 min; *solid bars*) inhibited both basal-CM and EGF-CM-induced migration compared with non-drug treated controls (p = 0.011 and p = 0.001, respectively), whilst the PI(3)Kγ-selective inhibitor, AS-252424 (30 μM, 30 min; *darker shaded bars*) significantly blocked EGF-CM-mediated chemotaxis (p = 0.021) (*n* = 8 donors). (B) Both basal-CM and EGF-CM–induced neutrophil apoptosis were markedly inhibited by LY294002 (30 μM; *solid bars*), but the PI(3)Kγ (AS-252424, 30 µM) and δ (IC87114, 10 µM; *hatched bars*)-selective inhibitors were partially effective (*n* = 9 donors). Data are mean ± SEM and statistical significance by Mann-Whitney test, **p<0.01 and ***p<0.001 *vs.* non-drug treated neutrophils.

We next studied whether activation of the myeloid-restricted class I PI(3)K isoforms, γ and δ which regulate fundamental cellular processes in neutrophils [39], [40], [41], [42] are involved in the regulation of chemotaxis induced by PBEC-CM from Mod/Sev asthmatics by applying each of the PI(3)K inhibitors at maximally effective concentrations [42]. Pre-treatment of neutrophils with the broad spectrum PI(3)K inhibitor, wortmannin, before exposure to basal-CM or EGF-CM blocked the neutrophil migration response (Mann-Whitney test p = 0.011 and p = 0.001, respectively) (Figure 5A). The PI(3)Kγ-selective inhibitor, AS-252424 reduced basal-CM–mediated chemotaxis (Mann-Whitney test p = 0.064) and significantly blocked the migratory response towards EGF-CM by 62.91% ±7.55% (Mann-Whitney test p = 0.021). In contrast, the PI(3)Kδ-selective inhibitor, IC87114 displayed no inhibitory effects (see Figure S1, in File S1), suggesting that PI(3)Kδ is not required to drive neutrophil chemotaxis in response to the generated mediators in PBEC-CM.

LY294002, a non-selective class I PI(3)K inhibitor with a longer half-life in culture than wortmannin was tested in neutrophil apoptosis assays *in vitro*. Epithelium–mediated neutrophil survival was wholly PI(3)K-dependent as seen by the complete reversal by LY294002 of the anti-apoptotic effects induced by both basal-CM and EGF-CM from Mod/Sev asthmatics (both ANOVAs, p<0.001, Figure 5B). In contrast, the regulation of the pro-survival effects were not isoform-specific for either PI(3)K δ or γ as their respective isoform-selective inhibitors, IC87114 and AS-252424 both partially (yet significantly, post-hoc test p = 0.003 and p = 0.005 for basal-CM; p = 0.008 and p = 0.001 for EGF-CM, respectively) reduced the effects of basal-CM and EGF-CM on neutrophil apoptosis in a functionally redundant manner (Figure 5B). None of the PI(3)K inhibitors significantly affected constitutive neutrophil apoptosis, suggesting that the isolated neutrophils were in culture in an unprimed state.

**Figure 6:**
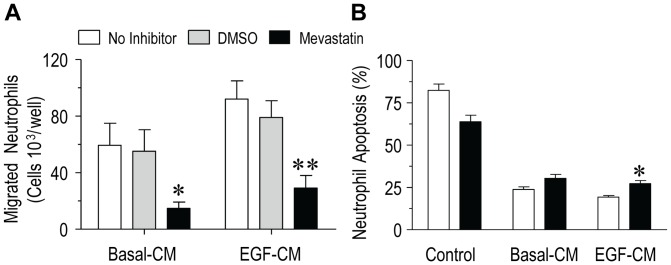
Regulation of RhoA GTPase during neutrophil chemotaxis and survival mediated by PBEC-CM from Mod/Sev asthmatics. (A) Pre-treatment of neutrophils with mevastatin (50 μM for 30 min; *solid bars*) attenuated both basal-CM and EGF-CM-mediated chemoattraction compared with the non-drug treated controls (p = 0.047 and p = 0.012, respectively). Vehicle-treated control cells were treated with 0.1% *v/v* DMSO (*n* = 5 donors). (B) Mevastatin did not affect basal-CM mediated neutrophil anti-apoptosis but partially inhibited EGF-CM-induced survival (p = 0.038) (*n* = 6 donors). Data represent mean ± SEM and statistical significance by paired *t*-test *p<0.05 *vs.* non-drug treated neutrophils.

We next investigated the regulatory role of RhoA GTPase on chemotaxis and pro-survival signaling mediated by PBEC-CM from Mod/Sev asthma patients. Treatment of neutrophils with the 3-hydroxy-3-methylglutaryl-coenzyme A (HMG-CoA) reductase inhibitor, mevastatin (50 μM, a published concentration, see Figure S2, File S1) diminished both basal-CM and EGF-CM–initiated chemoattraction from Mod/Sev PBECs (one-way ANOVA, p = 0.015 and p = 0.001; post hoc test, p = 0.047 and p = 0.012, respectively) (Figure 6A), suggesting potential coupling of the RhoA GTPase to neutrophil chemotactic responses. In contrast, pretreatment of neutrophils with mevastatin had a modest, but significant (paired *t*-test, p = 0.038, Figure 6B), effect on EGF-CM–induced anti-apoptotic activity, suggesting that neutrophil survival partially depends on RhoA signaling.

## Discussion

Employing an *ex vivo* model to study bronchial epithelial cell responses in asthmatics, the current study has made two novel important observations. First, EGF induced the production of pro-inflammatory mediators by epithelial cells from moderate to severe asthmatics that increased the chemotaxis and prolonged the survival of neutrophils. This pro-inflammatory activity was upregulated with increasing severity of asthma, pointing to an aberrant asthmatic bronchial epithelium as a potentially important source and further confirming its important role in promoting elevated neutrophil pro-survival activity detected in the airway epithelial lining fluid (induced sputum) in a subset of severe asthmatics with airway neutrophilia [4]. The second novelty comes from the observation that the epithelium acts on neutrophils via class I PI(3)K signaling. While the neutrophil migratory responses required class IB PI(3)Kγ signaling, the anti-apoptotic activity was dependent on multiple class I PI(3)Ks, suggesting differential regulation of these two mechanisms of cell accumulation. Although the current study was unable to identify any of the studied chemotactic candidates mediating neutrophil migration, the induced neutrophil survival response could be attributed to a significant extent to GM-CSF, a cytokine that is known to extend neutrophil longevity by delaying constitutive apoptosis [43], [44]. A summary of the potential interactions between neutrophils and an EGF-conditioned airway epithelium in enhancing neutrophil chemotaxis and pro-survival responses in asthma is provided in Figure S2 in File S1.

While previous studies have demonstrated that bronchial epithelial cells from normal and asthmatic donors generate pro-inflammatory mediators *ex vivo* that can influence neutrophil functional responses [14], [17], [43], [45], this study provides the first evidence that an EGF-induced airway epithelium can upregulate neutrophil accumulation in relation to asthma severity in a PI(3)Kγ dependent fashion. Using samples from relevant patient populations, the present translational findings provide a novel mechanistic perspective for neutrophilic mucosal inflammation that underpins the pathobiology of more severe phenotypes of asthma.

While both normal and asthmatic epithelia showed an ability to regulate neutrophil function, this was most pronounced in epithelium derived from asthmatic patients and following activation with EGF. Stimulation of PBECs from asthmatic patients with EGF induced significant up-regulation of such pro-neutrophilic factors as IL-6, IL-8, GM-CSF and TNF-α. This was seen in both MA and Mod/Sev patients except for IL-6 which was not upregulated in MA by EGF. The fact that only two of the four cytokines measured were upregulated in cultures from healthy subjects suggests a trend for EGF effects to be more pronounced in disease, especially in more severe asthma. Cytokine-neutralising experiments in Mod/Sev disease showed GM-CSF to be a contributor to the anti-apoptotic activity generated by EGF. This is consistent with enhanced GM-CSF levels observed in asthma-derived PBECs triggered by other relevant stimuli, such as mechanical wounding, pollutants and respiratory viruses [46], [47], [48].

In the current study, we also sought evidence for TARC/CCL17 and MDC/CCL22, two lymphotactic chemokines that are implicated in the recruitment of T lymphocytes and eosinophils, but these were either undetectable or low in most CM in all three subject groups. This suggests that the airway mucosa stimulated by EGF during epithelial injury and repair is likely to favour the pro-inflammatory potential of neutrophils rather than lymphocytes in the airways of more severe asthmatics. Furthermore, the biosynthetic capacity of the asthmatic PBECs to secrete these neutrophil-active factors remained preserved in culture even after the second passage *ex vivo*, reflecting an intrinsic secretory phenotype that exists *in vivo*. The evidence available from this and our previous study of primary human bronchial epithelial cells [14] suggests that EGF modulates the function of neutrophils via its action on the epithelium and not through a direct effect on neutrophils. The role of other EGFR ligands in relation to neutrophil function is unclear and will need to be elucidated; previous studies have shown that one of the EGFR ligands (HB-EGF) decreases neutrophil-endothelial interactions and can also block the generation of superoxide anions from human neutrophils [49], [50], [51].

We have previously shown that the asthmatic epithelium contains areas of exposed basal cells expressing EGFR on the luminal surface [19]. Our *ex vivo* model aimed to mimic activation of these cells *in vivo* by EGFR ligands present in the mucosal lining fluid. While others have used a scrape-wound model to examine responses in differentiated airway epithelial cells [52], such models are time-consuming and difficult to control (*e.g.* total *versus* partial thickness damage, size of the wound, basal activities of EGF *etc*). Our disease-related model provides a simple alternative that allows study of patient-derived epithelial cells with a basal cell phenotype and evaluation of mucosal inflammatory responses to such factors as EGF. Whilst the use of undifferentiated bronchial epithelial cells might also be seen as a limitation of the current study, this phenotype is appropriate for the aims of this study since it reflects an injured/repairing airway epithelium present in asthmatics *in vivo* [19]. Nevertheless, additional studies using differentiated primary epithelial cells grown under air-liquid interface would have been potentially valuable. This would have required identification of the most appropriate insult(s) (physical or chemical) that results in a dysregulated injury-repair process seen in asthma *in vivo* and this was beyond the scope of this manuscript. Thus, further studies investigating the potential neutrophil-modulatory capacity of pseudostratified epithelial cells established from asthmatic patients are needed.

The Rho family of small GTPases is a central regulator of the actin cytoskeleton in neutrophils during cell migration [53]. Selective inhibition of the HMG-CoA reductase by inhibitors, such as mevastatin, blocks the downstream biosynthesis of isoprenoid lipids, thus impairing RhoA GTPase membrane targeting/activation and consequent neutrophil-airway epithelial interactions [53], [54]. In agreement with this, we have found that blocking RhoA GTPase signaling in neutrophils with mevastatin led to a marked reduction of both basal-CM and EGF-CM–stimulated chemoattraction, suggestive of an involvement of RhoA signaling in the neutrophil chemotactic response.

Class I PI(3)Ks regulate leukocyte recruitment, activation and survival through the generation of PtdIns(3,4,5)P_3_ and are subdivided into class IA (PI(3)Kα, PI(3)Kβ and PI(3)Kδ) and class IB (PI(3)Kγ) [41]. However, how the different isoforms of these PI(3)Ks regulate epithelium-mediated neutrophil responses in clinical asthma is unknown. We focused our studies on the role of the myeloid-restricted PI(3)K γ and δ isoforms because of their proposed role in lung inflammation *in vivo* [33], [34], [35], [36]. Using a pharmacological approach to inactivate the catalytic domain of the p110γ, we demonstrated that PBEC-CM-mediated neutrophil migration was largely PI(3)Kγ-dependent (∼63%). Our findings are in agreement with previous studies using murine PI(3)Kγ knockouts and PI(3)Kγ selective inhibitors describing a major dependence upon class IB PI(3)Kγ signaling in neutrophil recruitment [39], [40], [55], particularly during pulmonary inflammation *in vivo* [33], [34], [56]. However, given the species (and tissue) differences and the inciting stimulus, it is important to exercise caution in translating observations from animal models to human clinical disease. For example, whereas primed oxidative burst in murine neutrophils is largely dependent on PI(3)Kγ alone, a biphasic control of both PI(3)Kγ and δ activation is required in regulating PtdIns(3,4,5)P_3_ signaling and consequent superoxide generation in human neutrophils [42]. This highlights the need to apply human-based *ex vivo* model systems to better understand PI(3)K signaling mechanisms in the disease context in order to develop isoform-selective anti-inflammatory therapeutics. In contrast, complete inhibition of neutrophil survival effect, (attributable in part to, GM-CSF, Fig 4B) was possible only with the non-selective PI(3)K inhibitor, LY294002, whereas the mono-selective γ and δ inhibitors were only partially effective. This suggests some functional redundancy amongst the PI(3)Ks in transducing the PBEC-CM mediated pro-survival signals. Consistent with this notion, pharmacological inhibition of at least three class I PI(3)K isoforms was shown to be required effectively to block GM-CSF-mediated survival effect in circulating neutrophils derived from both healthy individuals and acutely exacerbated patients with chronic obstructive pulmonary disease (COPD) [57].

The PI(3)Kδ-selective inhibitor, IC87114 failed to block neutrophil migration mediated by PBEC-CM from Mod/Sev asthmatics. This is consistent with results in chemokine and oxidants/cigarette smoke-induced neutrophil recruitment into the bronchoalveolar space in models of acute lung inflammation where IC87114 (or PI(3)Kδ-dead knock-in mice) was shown to be similarly ineffective when applied alone [34], [58], [59]. Given the possibility of functional redundancy of class I PI(3)Ks signaling in immune cells, one potential explanation is that PI(3)Kδ may play a more active role in driving the allergic-type inflammatory responses than independently regulating neutrophilic recruitment to inflammatory foci. Consistent with this notion, several pre-clinical studies using genetic and pharmacological inactivation of p110δ have described an isoform-specific role for PI(3)Kδ in regulating mast cell activation and degranulation [60], allergen-induced Th2 cytokine production and eosinophil recruitment into the inflamed airways *in vivo* [35], [36], [61] – immunological responses that are reminiscent of allergic asthma in humans [62]. In addition, significantly higher levels of PI(3)K 110δ mRNA expression (but not p110γ) are elevated in primary airway smooth muscle cells derived from patients with mild/moderate asthma compared to the non-asthmatic donors [63]. Of clinical relevance, a phase I clinical trial for an inhaled PI(3)Kδ-selective inhibitor is currently being undertaken to establish proof of mechanism in allergic asthma (http://clinicaltrials.gov/ct/show/NCT01462617), whilst CAL-101 (an analogue of IC87114) is being tested in patients with allergic rhinitis (http://clinicaltrials.gov/ct2/show/NCT00836914).

Our study has some limitations. Although the observed anti-apoptotic activity of the bronchial epithelial cells may prolong neutrophil survival, it is possible that alternative cell death programmes exist that are independent of apoptosis. Recent work has demonstrated non-apoptotic cell death pathways, such as oncosis/necrosis [64], [65], pyroptosis [65], cytolysis [66], NETosis [67] and autophagic-like neutrophil death mechanisms [68] that play important physiological roles in regulating neutrophil lifespan. In addition, the use of unprimed, blood-derived neutrophils from healthy donors raises the question of whether chemotactic and survival responses of neutrophils from asthmatic subjects might be different. However, neutrophil preparations from asthmatic patients often contain eosinophils and there is no purification method which can selectively deplete these granulocytes without priming/activating them. This study could have also applied molecular approaches to block the pro-neutrophil activities. However, we chose a direct, cytokine-neutralising approach to therapeutically mimic the anti-cytokine biologics that are currently undergoing clinical trials in uncontrolled severe asthma [69].

In conclusion, we have identified a potentially important regulatory mechanism whereby epithelial cells from patients with asthma, stimulated by EGF, a key regulator of airway mucosal pathobiology in asthma, influence neutrophil accumulation, possibly involving class IB PI(3)Kγ isoform signaling. The pro-survival activity for neutrophils increases with asthma severity, with evidence of GM-CSF playing a role and signaling *via* multiple class I PI(3)Ks in neutrophils. These data highlight the important functional regulation of class I PI(3)K isoforms in neutrophil function, with implications for the development of selective anti-inflammatory therapeutics for more severe forms of asthma.

## Supporting Information

File S1 **Supporting Methods. Figure S1**, Shows that the PI (3)Kδ-selective inhibitor, IC87114, (10 µM; *solid bars*) did not significantly (one way ANOVA, p = 0.16) affect either basal-CM and EGF-CM-directed neutrophil chemotaxis compared with the corresponding non-drug treated controls. Vehicle-treated control cells were treated with DMSO (0.1% *v/v*). Neutrophil chemotaxis was assessed using calcein-loaded cells in a fluorescence-based chemotaxis microplate. Data represent mean ± SEM from PBECs derived from *n* = 6 different Mod/Sev asthma patients and using peripheral-blood neutrophils from healthy subjects each performed in duplicate. **Figure S2, Schematic representation of potential regulation of neutrophil chemotactic and anti-apoptotic responses by an EGF-conditioned asthmatic epithelium**. Unresolved airway neutrophilia and disordered airway epithelial function are pathobiological features in more severe forms of asthma. It is proposed that an EGF-conditioned asthmatic epithelium modulates neutrophil migration *via* a potential signaling mechanism involving RhoA and class IB PI (3)Kγ signaling. EGF-conditioned epithelium also delays neutrophil constitutive apoptosis through the production of GM-CSF and via activation of all class I PI (3)Ks in neutrophils. **Table S1, Comparability of the absolute values representing neutrophil chemotactic and anti-apoptotic activity generated by PBEC-CM derived from patients with mild asthma, Mod/Sev asthma and healthy controls. Supporting References.**
(DOCX)
