# Supplementary material for: EGF-Induced Bronchial Epithelial Cells Drive Neutrophil Chemotactic and Anti-Apoptotic Activity in Asthma
Source: PLoS One. 2013 Sep 11;8(9):e72502. doi: 10.1371/journal.pone.0072502 (PMC3770689; doi:10.1371/journal.pone.0072502)
Supplement: File S1 — Supporting Methods. Figure S1, Shows that the PI (3)Kδ-selective inhibitor, IC87114, (10 µM; solid bars) did not significantly (one way ANOVA, p = 0.16) affect either basal-CM and EGF-CM-directed neutrophil chemotaxis compared with the corresponding non-drug treated controls. Vehicle-treated control cells were treated with DMSO (0.1% v/v). Neutrophil chemotaxis was assessed using calcein-loaded cells in a fluorescence-based chemotaxis microplate. Data represent mean ± SEM from PBECs derived from n = 6 different Mod/Sev asthma patients and using peripheral-blood neutrophils from healthy subjects each performed in duplicate. Figure S2, Schematic representation of potential regulation of neutrophil chemotactic and anti-apoptotic responses by an EGF-conditioned asthmatic epithelium. Unresolved airway neutrophilia and disordered airway epithelial function are pathobiological features in more severe forms of asthma. It is proposed that an EGF-conditioned asthmatic epithelium modulates neutrophil migration via a potential signaling mechanism involving RhoA and class IB PI (3)Kγ signaling. EGF-conditioned epithelium also delays neutrophil constitutive apoptosis through the production of GM-CSF and via activation of all class I PI (3)Ks in neutrophils. Table S1, Comparability of the absolute values representing neutrophil chemotactic and anti-apoptotic activity generated by PBEC-CM derived from patients with mild asthma, Mod/Sev asthma and healthy controls. Supporting References. (DOCX) [file pone.0072502.s001.docx]

**Supporting Information to**:

**EGF-induced Bronchial Epithelial Cells Drive Neutrophil Chemotactic and Anti-Apoptotic Activity in Asthma**

Mohib Uddin, ^1^ Laurie C. Lau, ^1^ Grégory Seumois, ^1^ Pandurangan Vijayanand, ^1^ Karl J. Staples, ^1^ Dinesh Bagmane, ^1^ Victoria Cornelius, ^1^ Paul Dorinsky, ^2^ Donna E. Davies ^1^ and Ratko Djukanović^1^

*^1^Academic Unit of Clinical and Experimental Sciences and the NIHR Southampton Respiratory Biomedical Research Unit, University of Southampton Faculty of Medicine, Sir Henry Wellcome Laboratories, Southampton University Hospital, Southampton, United Kingdom*

*^2^ GlaxoSmithKline, Respiratory Medical Development Center, Five Moore Drive, Research Triangle Park, Durham, USA.*

*Correspondence and reprint requests to:

Professor Ratko Djukanović

Academic Unit of Clinical and Experimental Sciences,

University of Southampton Faculty of Medicine,

Sir Henry Wellcome Laboratories,

Level F, South Block,

Southampton University Hospital,

Southampton, Hants.

United Kingdom, SO16 6YD.

E-mail: [R.Djukanovic@soton.ac.uk](mailto:R.Djukanovic@soton.ac.uk)

## **Supporting Methods**

***Subjects***

Twenty-eight non-smoking volunteers comprising of 11 moderate-to-severe asthmatics (Mod/Sev), 8 steroid-naïve mild asthmatics (MA) and 9 non-atopic healthy control (HC) subjects took part in the study after giving written informed consent (see Table 1). Subjects with mild asthma had symptoms >2x/week with forced expiratory volume in 1 sec (FEV_1_) >80% of predicted and used short-acting inhaled β_2_-agonists as needed for symptom relief. Subjects with moderate asthma were treated with low-dose inhaled glucocorticoids (GCs) and inhaled short-acting β_2_-agonists. Subjects with severe asthma were taking regular oral or high-dose inhaled GCs and a long-acting inhaled β_2_-agonist, and had FEV_1_ < 80% of predicted. The moderate and severe asthmatics were grouped into one group (Mod/Sev). Asthma severity was in keeping with Global Initiative for Asthma (GINA) 2010 criteria <http://www.ginasthma.org> (s1). Atopy was assessed by skin prick tests for common allergens. The age-matched healthy controls were non-atopic, had no history of smoking or respiratory symptoms suggestive of asthma and all had FEV_1_ >90% of predicted.

***Reagents and antibodies***

The PI(3)K inhibitors (wortmannin, LY294002, AS-252424 and IC87114) and the CXCR2 antagonist, SB-225002 were purchased from Calbiochem (Nottingham, UK). Mevastatin (Calbiochem) was used at a working concentration of 50 μM published previously (s2), where it specifically inhibited HMG-CoA reductase without exerting any non-specific effects on latter steps of cholesterol biosynthesis. Neutralizing monoclonal anti-TNF-α, GM-CSF and IL-6R antibodies were from R&D Systems (Abingdon, UK).

##### ***Primary bronchial epithelial cell culture from asthmatic patients and healthy controls***

PBEC cultures were established from endobronchial brushings as previously described (s3). Bronchial epithelial cells were cultured in hormonally supplemented bronchial epithelial cell basal medium (BEBM) (Clonetics, San Diego, CA, USA) in collagen-coated tissue culture flasks (Gibco-BRL, Paisley, UK) at 37°C in 5% (v/v) CO_2_. Submerged second passage PBECs (1 x 10^5^ cells/well) were seeded into collagen-coated 24-well plates and grown to 70% confluency. Cells were rendered quiescent in BEBM basal medium (Clonetics, USA), supplemented with 1% (v/v) insulin/transferrin/sodium selenite (Sigma, Poole, UK) and 0.3% (v/v) bovine serum albumin (BSA) for 24 h. For epithelial cell activation, PBECs were incubated with EGF (10 ng/ml, a working concentration that has no direct effects on neutrophil function (s3)) or serum-free medium (SFM) for 24 h; this generated EGF-conditioned media (EGF-CM) and basal-conditioned media (basal-CM), respectively.

***Assessment of bronchial epithelial cell proliferation in response to EGF***

Bronchial epithelial cell numbers were determined by uptake of methylene blue as previously described (s4 – s7). Subconfluent epithelial cells were seeded into 24-well plates at 2.5 x 10^4^ cells/well in complete BEGM medium (Lonza, Workingham, UK) and cultured at 37°C for 6 h. The medium was changed to serum-free medium (SFM), serum starved overnight, and then stimulated with SFM or EGF (10 ng/ml) for 24 h. After treatment, the medium was removed and the cells were fixed with 4% formaldehyde in 0.9% NaCl solution at 20°C for 1 h. The fixed cells were stained with 1% methylene blue in 10 mM disodium tetraborate (pH 8.5) for 30 min and then washed again with the same borate buffer. Bound methylene blue was eluted with 1% HCl in ethanol and cell number determined by measuring the absorbance at 650 nm (A_650_) with a microplate spectrophotometer (MultiScan Ascent, Affinity Sensors, Cambridge, UK). A_650_ was directly proportional to the epithelial cell number over the range of cell densities. The stimulation index was calculated by dividing the mean number of epithelial cells exposed to EGF (10 ng/ml, 24 h) by the mean number of cells incubated with SFM alone.

***Isolation of neutrophils from healthy human volunteers***

Peripheral blood neutrophils were obtained from healthy non-medicated adult donors and purified using dextran sedimentation and discontinuous plasma-Percoll gradients as previously described (s3). Neutrophils were carefully harvested from the 42%/51% Percoll interface then were washed sequentially with Dulbecco’s PBS without and with Ca^2+^ and Mg^2+^. Neutrophil purity, assessed by cytospin was routinely **>**96% with viability **>**98% as assessed by trypan blue exclusion.

***Assessment of neutrophil chemotaxis***

Neutrophil chemotaxis was assayed using ChemoTx^©^ microplates (Neuroprobe, Receptor Technologies, Gaithersburg MD, USA), as previously detailed (s3). Briefly, purified neutrophils (1 × 10^7^ cells/ml) were incubated with 5 μM calcein in HBSS supplemented with 10% autologous serum for 45 min at 37°C. The lower wells of the microplate were filled in duplicate with either 31 µl of assay buffer, SFM, basal-CM or EGF-CM derived from asthmatic and healthy control PBECs. Calcein-labeled neutrophils (3 × 10^5^ cells/ml) were loaded to the upper aspect of the membrane and allowed to migrate for 60 min (37°C in 5% CO_2_). For inhibition studies, neutrophils were pretreated with the putative inhibitors for 30 min prior to addition to the membrane. Non-migrated cells were aspirated and detachment buffer (PBS with 20 mM EDTA and 0.3% BSA v/v) was applied on the membrane and incubated for 30 min at 4°C followed by flushing with PBS and centrifugation. The membrane was removed and the numbers of transmigrated neutrophils in the lower wells were quantified by measuring fluorescence (485 nm excitation, 530 nm emission wavelengths) using a fluorescence plate reader (FLX 800 Microplate; Bio-Tek, Winooski, VT, USA). Of note, due to limited amounts of asthmatic PBEC-derived conditioned media, additional experiments were not performed to distinguish neutrophil chemotaxis from chemokinesis. Whilst we acknowledge that chemoattractants can also promote chemokinesis, our past studies, during which the methods for investigating chemotaxis were developed, have shown that the majority of the activity measured using complex conditioned media or samples of the airways lining fluid is chemotactic rather than chemokinetic. Consequently, all our previous studies on this topic have only focused on chemotaxis (s8 – s13).

***Flow cytometry analysis of neutrophil apoptosis***

Purified neutrophils were resuspended at 5 x 10^6^ cells/ml in Iscove’s DMEM supplemented with 10% autologous serum and 50 U/ml streptomycin and penicillin G for 20 h at 37°C in 5% CO_2_. PBEC-CM derived from asthmatic patients or healthy control subjects were incubated with the purified neutrophils for 20 h *in vitro*. Neutrophils were harvested by centrifugation at 300 *g* for 5 min at 4^o^C, washed in PBS then resuspended in Annexin-V binding buffer (10 mM HEPES, pH 7.4, 140 mM NaCl, 2.5 mM CaCl_2_) containing fluorescein isothiocyanate (FITC)-labeled Annexin-V (1 μg/ml) and propidium iodide (12.5 μg/ml). Annexin-V^FITC^/PI-stained cells were incubated for 15 min at 4°C in the dark and then diluted with 100 µl of binding buffer prior to analysis by flow cytometry using a FACSCalibur with Cell Quest software (BD Biosciences, Oxford, U.K) by measuring the relative fluorescence of 10,000 gated neutrophils per sample. Apoptotic neutrophils were identified as positive for Annexin-V^FITC^ and negative for PI (Annexin-V^FITC+^/PI^−^) whilst viable, non-apoptotic neutrophils were defined as negative for both Annexin-V^FITC^ and PI (Annexin-V^FITC−^/PI^−^). Neutrophil apoptosis was expressed as a percentage of apoptotic cells (Annexin-V^FITC+^/PI^−^) in relation to the total number of counted neutrophils.

**Supporting Results & Figures**

**Figure S1**

***Fig. S1****.* shows that the PI(3)Kδ-selective inhibitor, IC87114, (10 µM; *solid bars*) did not significantly (one way ANOVA, p=0.16) affect either basal-CM and EGF-CM-directed neutrophil chemotaxis compared with the corresponding non-drug treated controls. Vehicle-treated control cells were treated with DMSO (0.1% *v/v*). Neutrophil chemotaxis was assessed using calcein-loaded cells in a fluorescence-based chemotaxis microplate. Data represent mean ± SEM from PBECs derived from *n* = 6 different Mod/Sev asthma patients and using peripheral-blood neutrophils from healthy subjects each performed in duplicate.

**Figure S2**

***
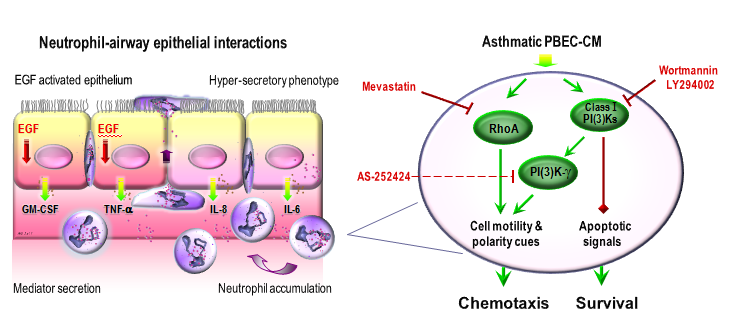
***

***Fig. S2****.* **Schematic representation of potential regulation of neutrophil chemotactic and anti-apoptotic responses by an EGF-conditioned asthmatic epithelium**.

Unresolved airway neutrophilia and disordered airway epithelial function are pathobiological features in more severe forms of asthma. It is proposed that an EGF-conditioned asthmatic epithelium modulates neutrophil migration *via* a potential signaling mechanism involving RhoA and class IB PI(3)Kγ signaling. EGF-conditioned epithelium also delays neutrophil constitutive apoptosis through the production of GM-CSF and via activation of all class I PI(3)Ks in neutrophils.

***Table S1***. **Comparability of the absolute values representing neutrophil chemotactic and anti-apoptotic activity generated by PBEC-CM derived from patients with mild asthma, Mod/Sev asthma and healthy controls**

| **Chemotaxis**  *(Neutrophils 10^3^/well)* | | **Healthy Controls** | | **Mild Asthmatics** | | **Moderate/Severe Asthmatics** | |
| --- | --- | --- | --- | --- | --- | --- | --- |
| *Fig. 2A* | **SFM** | **Basal-CM EGF-CM** | | **Basal-CM EGF-CM** | | **Basal-CM EGF-CM** | |
| ***n*** | 8 | 8 | 8 | 8 | 8 | 8 | 8 |
| **Mean ± S.D.** | 19.21 ± 18.44 | 50.78 ± 42.55 | 69.57 ± 34.69 | 85.13 ± 37.20 | 106.12 ± 27.63 | 63.52 ± 37.91 | 124.69 ± 50.77 |
| **Mean Difference (95% CI)** | *n/a* | 18.79  (3.81, 33.84) | | 20.99  ( -4.53, 46.49) | | 61.17  ( 19.48, 102.13) | |
| **p value**  *(Basal vs EGF)* | *n/a* | 0.021* | | 0.093 | | 0.010* | |

| **Neutrophil Apoptosis *%*** | | **Healthy Controls** | | **Mild Asthmatics** | | **Moderate/Severe Asthmatics** | |
| --- | --- | --- | --- | --- | --- | --- | --- |
| *Fig. 2B* | **SFM** | **Basal-CM EGF-CM** | | **Basal-CM EGF-CM** | | **Basal-CM EGF-CM** | |
| ***n*** | 6 | 7 | 7 | 7 | 7 | 7 | 7 |
| **Mean ± S.D.** | 60.31 ± 16.80 | 54.70 ± 23.83 | 61.38 ± 22.86 | 38.02 ± 17.77 | 44.27 ± 21.71 | 32.16 ± 16.64 | 29.92 ± 15.35 |
| **Mean Difference (95% CI)** | *n/a* | 6.68  (-19.24, 32.52) | | 6.25  (-3.59, 16.10) | | -2.24  (-11.57, 7.22) | |
| **p value**  *(Basal vs EGF)* | *n/a* | 0.221 | | 0.171 | | 0.583 | |

**Supporting References**

(s1). **Global Initiative for Asthma (GINA) 2010**: Global Strategy for Asthma Management and Prevention. Available from: <http://www.ginasthma.org>

(s2). **Corsini A**, Maggi FM, Catapano AL. Pharmacology of competitive inhibitors of HMG-CoA reductase. *Pharmacol Res* 1995;31:9-27.

(s3). **Uddin M**, Seumois G, Lau LC, Rytila P, Davies DE, Djukanovic R. Enhancement of neutrophil function by the bronchial epithelium stimulated by epidermal growth factor. *Eur Respir J* 2008;31:714-724.

(s4). **Oliver MH**, Harrison NK, Bishop JE, Cole PJ, Laurent GJ. A rapid and convenient assay for counting cells cultured in microwell plates: Application for assessment of growth factors. *J Cell Sci* 1989;92 (Pt 3):513-518.

(s5). **Richter A**, Puddicombe SM, Lordan JL, Bucchieri F, Wilson SJ, Djukanovic R, Dent G, Holgate ST, Davies DE. The contribution of interleukin (IL)-4 and IL-13 to the epithelial-mesenchymal trophic unit in asthma. *Am J Respir Cell Mol Biol* 2001;25:385-391.

(s6). **Hamilton LM**, Torres-Lozano C, Puddicombe SM, Richter A, Kimber I, Dearman RJ, Vrugt B, Aalbers R, Holgate ST, Djukanović R, Wilson SJ, Davies DE. The role of the epidermal growth factor receptor in sustaining neutrophil inflammation in severe asthma. *Clin Exp Allergy* 2003;33(2):233-40.

(s7). **Davies ER,** Haitchi HM, Thatcher TH, Sime PJ, Kottmann RM, Ganesan A, Packham G, O'Reilly KM, Davies DE. Spiruchostatin A inhibits proliferation and differentiation of fibroblasts from patients with pulmonary fibrosis. *Am J Respir Cell Mol Biol*. 2012;46(5):687-94.

(s8). **Louis R**, Shute J, Biagi S, Stanciu L, Marrelli F, Tenor H, Hidi R, Djukanovic R. Cell infiltration, ICAM-1 expression, and eosinophil chemotactic activity in asthmatic sputum. *Am J Respir Crit Care Med* 1997; 155:466-472.

(s9). **Hidi R,** Riches V, Al-Ali M, Cruikshank WW, Center DM, Holgate ST, Djukanovic R. The role of B7-CD28/CTLA-4 co-stimulation and NF-kB in allergen-induced T cell chemotaxis by interleukin-16 and RANTES. *J Immunol* 2000; 164:412-418.

(s10). **Dent G**, Hosking LA, Lordan JL, Steel MD, Cruikshank WW, Center DM, Ellis JH, Holgate ST, Davies DE, Djukanovic R. Differential roles of IL-16 and CD28/B7 costimulation in the generation of T-lymphocyte chemotactic activity in the bronchial mucosa of mild and moderate asthmatic individuals. *J Allergy Clin Immunol* 2002; 110(6):906-14.

(s11). **Dent G**, Hadjicharalambous C, Yoshikawa T, Handy RC, Powell J, Anderson IK, Davies D, Louis R, Djukanovic R. Contribution of eotaxin-1 to eosinophil chemotactic activity of moderate and severe asthmatic sputum. *Am J Resp Crit Care Med*; 2004; 169: 1110-7.

(s12). **Yoshikawa T**, Dent G, Ward J, Angco G, Nong G, Hirata K, Djukanovic R. Impaired neutrophil chemotaxis in chronic obstructive pulmonary disease (COPD). *Am J Respir Crit Care Med* 2006; 2004;169:1110-7.

(s13). **Vijayanand P**, Durkin K, Hartmann G, Morjaria J, Seumois G, Staples KJ, Hall D, Bessant C, Bartholomew M, Howarth PH, Friedmann PS, Djukanovic R. Chemokine receptor 4 plays a key role in T cell recruitment into the airways of asthmatic patients. *J Immunol*. 2010;184 (8):4568-74.
